# Mechanochemical Synthesis of TiO_2_-CeO_2_ Mixed Oxides Utilized as a Screen-Printed Sensing Material for Oxygen Sensor

**DOI:** 10.3390/s23031313

**Published:** 2023-01-24

**Authors:** Jelena N. Stevanović, Srđan P. Petrović, Nenad B. Tadić, Katarina Cvetanović, Ana G. Silva, Dana Vasiljević Radović, Milija Sarajlić

**Affiliations:** 1 Institute of Chemistry, Technology and Metallurgy, University of Belgrade, Njegoševa 12, 11000 Belgrade, Serbia; 2 Faculty of Physics, University of Belgrade, Studentski trg 16, 11000 Belgrade, Serbia; 3 CeFiTec, Nova School of Science and Technology, New University of Lisbon, Campus da Caparica, 2829-516 Caparica, Portugal

**Keywords:** combustion control, oxygen sensing, TiO_2_-CeO_2_ mixtures, mechanochemistry, screen-printing, thick films, mixed-oxides-based sensor

## Abstract

TiO_2_ and CeO_2_ are well known as oxygen sensing materials. Despite high sensitivity, the actual utilization of these materials in gas detection remains limited. Research conducted over the last two decades has revealed synergistic effects of TiO_2_-CeO_2_ mixed oxides that have the potential to improve some aspects of oxygen monitoring. However, there are no studies on the sensing properties of the TiO_2_-CeO_2_ obtained by mechanochemical treatment. We have tested the applicability of the mechanochemically treated TiO_2_-CeO_2_ for oxygen detection and presented the results in this study. The sensing layers are prepared as a porous structure by screen printing a thick film on a commercial substrate. The obtained structures were exposed to various O_2_ concentrations. The results of electrical measurements showed that TiO_2_-CeO_2_ films have a significantly lower resistance than pure oxide films. Mixtures of composition TiO_2_:CeO_2_ = 0.8:0.2, ground for 100 min, have the lowest electrical resistance among the tested materials. Mixtures of composition TiO_2_:CeO_2_ = 0.5:0.5 and ground for 100 min proved to be the most sensitive. The operating temperature can be as low as 320 °C, which places this sensor in the class of semiconductor sensors working at relatively lower temperatures.

## 1. Introduction

The increase in greenhouse gases emission began with the industrial revolution and continues to the present day [1]. As fossil fuel combustion represents the main contribution to CO_2_ emission, combustion control in transportation and industry has become a top priority in modern environmental protection [1,2]. Novel environmental regulations have encouraged the development and testing of gas-sensitive materials for on-site monitoring of combustion in power plants, heating systems, transportation, and industry [3]. The optimization of exhaust gas composition during combustion increases the efficiency of the process via feedback systems and eventually leads to the reduction of a carbon footprint and lower fuel consumption [2,4]. Controlled combustion conditions in automotive applications and industrial plants can be maintained using sensors for monitoring exhaust oxygen concentration at elevated temperatures [3].

Several types of oxygen sensors have been developed, depending on the sensing mechanism and active material, such as optical sensors [5], semiconductor metal oxide sensors [6], electrochemical sensors [6], cantilever-based sensors [7], etc. Semiconductor metal oxide gas sensors belong to the group of the most investigated gas sensors [8]. Simple design, miniature packaging, low cost, and facile fabrication [9] make them suitable for integration into sensor networks for air-quality monitoring [7].

Properties and applications of, separately, TiO_2_ and CeO_2_ are well known and extensively investigated [10,11,12]. Many studies have proven the great oxygen sensing features of TiO_2_ and CeO_2_ thin films at 600 °C, 700 °C, and 800 °C [13,14,15,16]. At lower temperatures, thermodynamic equilibrium on the solid-gas interface between the sensing layer and oxygen cannot be achieved [17]. However, operating at high temperatures may lead to a decrease in the long-term stability of the sensor [6]. Insufficient long-term stability represents one of the limiting factors in the application of sensors based on metal oxide [3]. One way to overcome temperature-related disadvantages and improve stability is by adding a metal oxide to the primary oxide or by direct synthesis of mixed metal oxides [6,18].

In the last two decades, TiO_2_-CeO_2_ mixed systems have become an increasingly common topic of research in the field of gas sensing and Materials Science [19,20,21]. Mohammadi and Fray [22] studied the sensing properties of TiO_2_-CeO_2_ thin films obtained by the aqueous sol–gel route. They showed the remarkable response of a thin film-based sensor towards low concentrations of CO gas at the operating temperature of 200 °C. The use of sol-gel prepared TiO_2_-CeO_2_ thin films in gas sensing applications was also investigated by Trinchi and his coworkers [23]. The sensor response was measured on operating temperatures below 470 °C and for oxygen concentrations between 1 ppm and 10,000 ppm. It was found that the sensors based on materials with excess CeO_2_ have higher sensitivity than others. In 2016, Uzunoglu et al. [24] reported an increasing oxygen storage capacity when mixing TiO_2_ and CeO_2_ using the coprecipitation method. These reports [23,24] also point out the importance of the composition of composites, i.e., the mass ratios of starting oxides, for reliable oxygen detection. However, there is no work examining the influence of dry mechanochemical treatment on the oxygen sensing properties of TiO_2_-CeO_2_ mixtures. The possibility of using mechanochemically activated TiO_2_-CeO_2_ mixtures as sensing material for improving the performance of oxygen sensor is thus unexplored.

Mechanochemical preparation of powder mixture has recently emerged as a cost-effective and fast technology method for obtaining an active catalyst with excellent structural and morphological properties [25,26,27]. As a treatment that does not require toxic metal-organic precursors nor leaves harmful waste, it has no environmental footprint [25,26,27,28,29,30,31]. High-energy ball milling is a mechanochemical process that involves the direct absorption of mechanical energy by solid reactants. Energy is being released during collision, shear, and friction between the balls and the reactants inside the jar, and it is sufficient to initiate and stimulate chemical reactions. Intense mechanical stresses and bond breakage are induced by the effect of mechanochemical forces, leading to structural changes and the increasing reactivity of atoms at the reactant interface [31,32].

We conducted systematic testing of TiO_2_-CeO_2_ mixtures obtained by high energy ball milling and deposited by screen-printing of the paste on commercial alumina substrate with a gold interdigitated capacitor. Screen-printed thick TiO_2_-CeO_2_ films were then exposed to different partial pressures of O_2_ in the flow of the Ar-O_2_ gas mixture. In order to evaluate the sensitivity of our materials at lower operating temperatures, sensor response measurements were carried out in the temperature range from 270 °C to 400 °C. At the starting operating temperature, the electrical readout proved to be very unstable. A temperature of 320 °C was the minimum operating temperature at which response stability was satisfactory, and oxygen sensitivity was high. Therefore, measurements of the electrical resistance of the sensors performed at 320 °C are presented. Particle size distribution analysis (PSD), X-ray diffraction (XRD), scanning electron microscopy (SEM), atomic force microscopy (AFM), and Raman spectroscopy were employed to study the morphological, structural, and electronic properties of the obtained materials. In order to provide an understanding of sensing performance of the mixtures, the results of these method of characterizations were discussed.

## 2. Materials and Methods

### 2.1. Mechanochemical Synthesis of TiO_2_-CeO_2_ Powders

**Figure 1:**
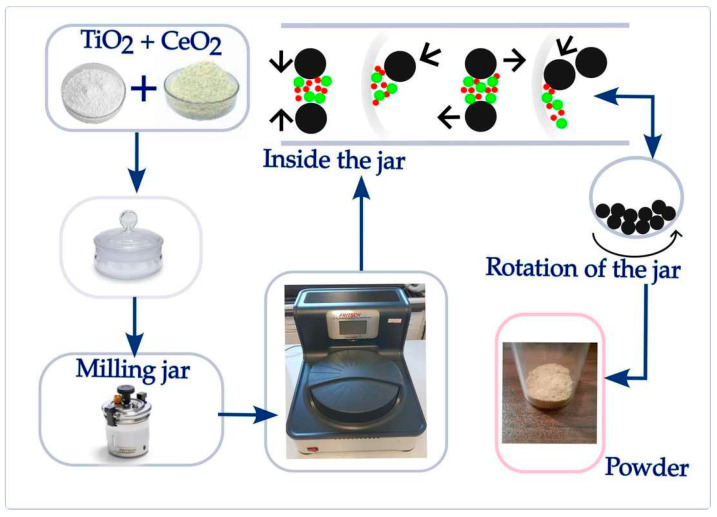
Schematic illustration of the mechanochemical procedure. Also shown are the processes that occur during high ball milling inside the jar: collision, impact, shear, and friction.

Dry mechanochemical synthesis of TiO_2_-CeO_2_ mixtures was performed using a high-energy ball mill (Planetary Micro Mill Pulverisette 7 premium line, Fritsch, Germany). The synthesis procedure is illustrated in Figure 1.

Pure TiO_2_ (purity > 99%, purchased, Alfa Aesar, ThermoFisher GmbH, Kandel, Germany) and CeO_2_ (purity >99.9, purchased, Johnson Matthey Alfa Product, Karlsruhe, Germany) powders were used as a starting material. Milling was conducted at atmospheric conditions in a silicon nitride jar, 80 cm^3^ in volume, with 25 silicon nitride balls with a diameter of 10 mm. The ball-to-total powder mass ratio was constant at about 1:10 for all the experiments. The quantity of powder mixture used in each synthesis was 4.87 g, with the following mass ratios of TiO_2_ to CeO_2_: 0.8:0.2, 0.2:0.8, and 0.5:0.5. The mixtures were grounded for either 40 or 100 min with a 5-min break after every 20 min of the process. The rotation speed of a milling jar was kept constant at 350 rpm during all mixture preparations. A total of six different powders were obtained. The labeling of the mixed oxides according to the grinding conditions is shown in Table 1. The obtained powders were investigated with the help of particle size distribution analysis and X-ray diffraction. Thick films representing sensing materials are made from powders by a procedure that will be described below.

#### 2.1.1. Particle Size Distribution Analysis

The high-energy shear forces between milling balls in most cases facilitate particle size reduction and improve the surface properties of the powders [32,33]. In order to evaluate the influence of different milling times on the average particle size of powders and their size distribution, particle size analysis (PSA) was employed. The used instrument was the Mastersizer 2000 (Malvern Panalytical, Malvern, UK) particle size analyzer based on laser diffraction, which covers the particle size range of 0.02-2000 µm. For the sake of the PSA measurements, the powders were dispersed in ethanol and treated in an ultrasonic bath, low-intensity ultrasound, at a frequency of 40 kHz and power of 50 W.

**Figure 2:**
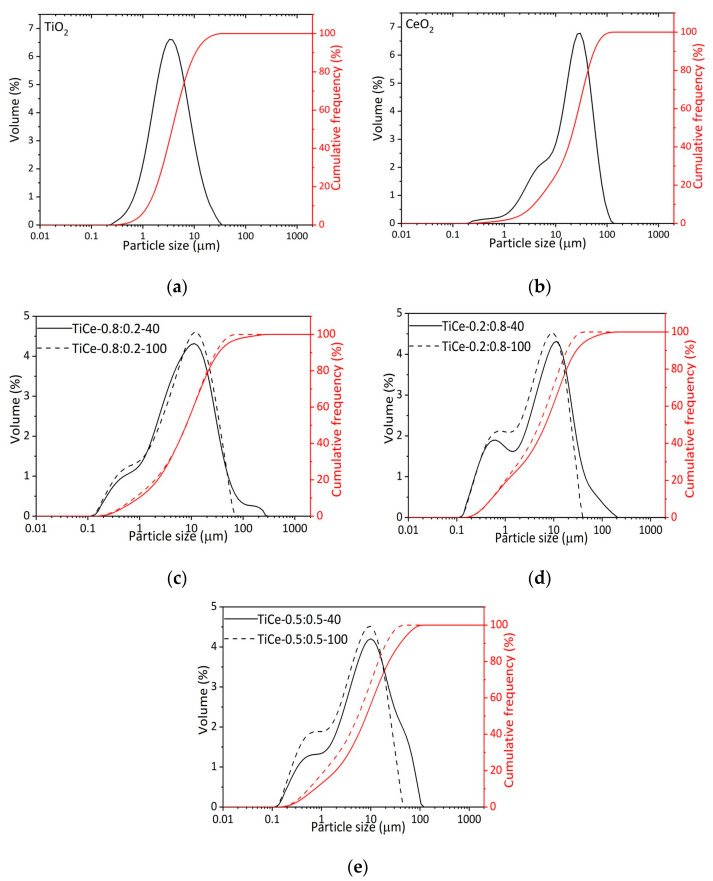
Volume-based particle size distribution and cumulative frequency of: (**a**) TiO_2_; (**b**) CeO_2_; (**c**–**e**) mixed oxide powders.

In Figure 2c–e the effect of ball milling treatment on particle size distribution is presented. The particle size distribution of starting oxides is also given (Figure 2a,b). The following characteristic diameters are shown: d_10_ (10% of particles smaller than this diameter), d_50_ (average particle size), and d_90_ (90% of particles smaller than this diameter) [34]. It is noticeable that mixtures grounded for 40 min (TiCe-0.8:0.2-40, TiCe-0.2:0.8-40, TiCe-0.5:0.5-40) have a larger average particle size (d_50_) and wider particle size distribution than those grounded for 100 min (TiCe-0.8:0.2-100, TiCe-0.2:0.8-100, TiCe-0.5:0.5-100). The width of the distribution is usually quantified by calculating the span as (d_90_-d_10_)/d_50_ [34].

The smallest average particle sizes were observed for TiCe-0.2:0.8-100 and TiCe-0.5:0.5-100 samples, 5.01 µm and 5.50 µm, respectively. The distribution curves of mixtures ground for 100 min are characterized by the absence of a tail corresponding to the size of particles larger than 100 µm (Figure 2c–e), which leads to the conclusion that a prolonged mechanochemical process promotes the fragmentation of agglomerates. Considering the values of average particle size previously mentioned, it can be assumed that the influence of mechanical forces on the particle size is more pronounced for mixed oxides with a high amount of CeO_2_, i.e., TiCe-0.2:0.8-100 and TiCe-0.5:0.5-100. A possible explanation lies in the significant difference between the particle size distribution curve of starting oxides. In fact, TiO_2_ particle size values at 10%, 50%, and 90% of the cumulative frequency are less than those of CeO_2_. The particle size values at 10%, 50%, and 90% of the cumulative weight and span values are shown in Table 2.

#### 2.1.2. X-ray Powder Diffraction

X-ray powder diffraction (XRPD) was used to examine changes in the crystal structure of the powders after undergoing mechanochemical treatment. Diffraction data were acquired over the scattering angle from 20° to 70° with a speed of 0.05 °/s and an acquisition time of 1 min using the Rigaku Ultima IV diffractometer in Bragg-Brentano geometry (Tokyo, Japan). The irradiation used was Ni-filtered CuKα radiation with a wavelength of 1.542 Å.

**Figure 3:**
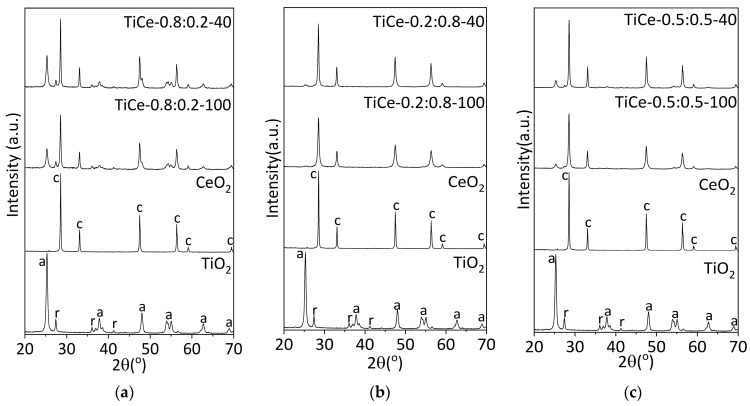
XRD patterns of starting oxides and mixtures of the following composition: (**a**) TiO_2_:CeO_2_ = 0.8:0.2; (**b**) TiO_2_:CeO_2_ = 0.2:0.8; (**c**) TiO_2_:CeO_2_ = 0.5:0.5; a—anatase, r—rutile, c—ceria fluorite structure.

Figure 3a–c displays the XRPD patterns of TiO_2_-CeO_2_ mixed oxides and starting powders. The results are arranged in order to visualize the influence of the milling time. XRPD measurements provide characteristic reflections of the anatase and rutile phases of TiO_2_ and the cubic form of CeO_2_. No reflections belonging to the new crystalline structures are present in the TiO_2_-CeO_2_ patterns. This indicates that there is no detectable chemical reaction between TiO_2_ and CeO_2_ in composites [35]. The absence of crystalline Ce-Ti-O compounds could be a consequence of the method of preparation of mixed oxides and the preparation conditions: composition, temperature, and atmosphere [36,37]. The formation of various compounds with Ce-Ti-O interaction, such as Ce_2_TiO_5_, Ce_2_Ti_2_O_7_, and Ce_4_Ti_9_O_24_, has been reported in the literature when heating appropriate mixtures to a temperature of 1523 K [36]. The mechanical energy and local temperature introduced into the system under the given milling conditions are insufficient to cause a chemical reaction that leads to the formation of more complex oxides.

The mechanochemical treatment did not affect the position of the peak in any of the presented patterns. Shifting of the peak would indicate a change in the lattice parameters as a result of the incorporation of the dopant atoms into the host lattice [38]. This further confirms that the unit cell parameters remained unchanged, and no solid solution was formed.

The phase crystallite size in the mixtures was evaluated and summarized in Table 3. The most prominent reflection of the mixed oxides tended to increase the full width at half maximum (FWHM) compared to the peaks of the pure components. The broadening of diffraction peaks is a measure of deviation from the ideal crystal structure, such as the final crystallite size, and lattice strain arising from increased dislocations and two-dimensional lattice defects [39,40]. Increasing the milling time led to a further broadening of reflection peaks and a weakening of its intensity, indicating a reduction in crystallite size and a decrease in the amount of the corresponding phase [26]. The average crystallite size (D) was estimated from the full width at half maximum of the most prominent peak using the Scherrer formula, ignoring the contribution of strain [38]:

D = Kλ/βcosθ(1)

Here, K is the morphological parameter for spherically shaped particles equal to 0.94 [26], λ is the wavelength of the radiograph (1.542 Å) for CuK_α_ radiation, θ is the Bragg diffraction angle, and FWHM is rewritten as β and expressed in radians. Another consequence of subjecting the crystal to mechanochemical actions is the microstrain of the crystal lattice. Therefore, it is recommended to use the Warren-Averbach or Williamson–Hall methods [41] to estimate the effects of crystallite strain and crystallite size on the XRD peak width, separately. However, considering that ceramic materials are weakly influenced by strain hardening, even when containing dislocations [42], the use of Scherrer equations is justified.

As it can be noted from Table 3, the mean crystallite size of anatase and ceria phases in mechanochemically treated mixtures decreases with increasing milling time (mark “-“ signifies the absence of a reflection of the corresponding phase in the spectrum). It should be stressed that this trend was not observed in the case of the rutile phase. Comparing the post-synthesis crystallite size to that of the pure TiO_2_ and CeO_2_, it is observed that prolonged milling time affects the CeO_2_ crystallite size to a greater extent than anatase, in all mixtures except the one labeled as TiCe-0.2:0.8-40. Crystal size depends not only on the milling time but also on the composition of the mixture. The estimated values indicate that with an increase in the amount of CeO_2_ and, at the same time, a decrease in the amount of TiO_2_ in the mixture, the crystallite size of both mentioned phases reduces. The largest reduction in the size of CeO_2_ crystallites was observed in the sample milled for 100 min and containing 80% CeO_2_ (TiCe-0.2:0.8-100). Weak or absent reflection of TiO_2_ in the XRD pattern of the aforementioned sample (Figure 3b) suggests a decrease in crystallite size to the extent that it cannot be detected under the given conditions.

The intensity of rutile reflection in the XRPD pattern of milled powders varies depending on the milling time and the composition of the mixture (Figure 3a–c). Rutile phase fraction was determined by using the anatase (101) and rutile (110) peak, according to Spurr’s equation [26]:

f = 1−1/(1 + 1.265I_r_/I_a_) (2)

where Ir and Ia represent the peak intensities of rutile and anatase, respectively. The calculated values listed in Table 4 show that all milled powders contain a larger amount of the rutile phase with respect to TiO_2_. The rutile weight fraction in mixtures of the same composition increases with prolonged milling time.

Due to the friction, shear, and collisions between the milling balls and the powder, so-called “hot spots” are formed, which are characterized by a rise in temperature and pressure [30,43]. Local high temperature and pressure induce anatase to rutile transition [26]. The change in the weight fraction of the phase in the mixtures with the change in the synthesis conditions might be the reason for the observed increase in the size of the rutile crystallites with the increase in milling duration.

### 2.2. TiO_2_-CeO_2_ Paste Preparation and Sensor Fabrication

The TiO_2_-CeO_2_ pastes were prepared by following the recipe for the fabrication of TiO_2_ paste for dye-sensitized solar cells, which was established by Ito and his coworkers [44]. A mass of 1 g of mechanochemically treated powders was ground in a porcelain mortar with the addition of acetic acid, water, and ethanol, respectively. The content of the dispersion in ethanol was concentrated by evaporating ethanol in a desiccator on a magnetic stirrer. The pressure was lowered to 10 mbar using a vacuum pump. The same procedure was applied for making TiO_2_ and CeO_2_ pastes from starting powders.

**Figure 4:**
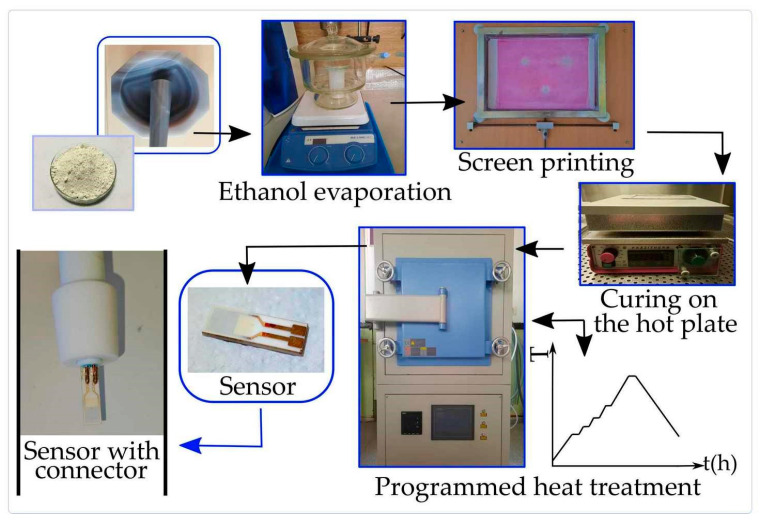
Schematic illustration of paste preparation and sensor fabrication.

The step-by-step sensor fabrication is illustrated in Figure 4. Before applying each layer, the paste is homogenized by stirring on a magnetic stirrer (MR 3001 K, Heidolph, Wood Dale, IL USA). A total of six layers are deposited by a screen-printing technique on an alumina substrate, in the form of thick films. Commercial substrates DropSense InterDigitated Gold Electrodes with a Platinum heater on the other side (DropSense, Interdigitated Gold Electrode/200 microns lines and gaps, Metrohm AG, Herisau, Switzerland) were used. Screen printing equipment used in this work was handmade. After each layer application, the substrate was dried on a hot plate (PZ28-2, Präzitherm, Harry Gestigkeit GmbH, Düsseldorf, Germany) at 125 °C for 6 min. The last step in sensor fabrication is programmed heat treatment in an oven (CY-A1200-4IT, Zhengzhou CY Scientific Instrument Co., Ltd., Zhengzhou, China) up to 800 °C in an atmosphere of Ar. The heating process serves to degrade the organic substances from the paste and sinter the sensing layer during the temperature hold. By initiating the necks growth between the particles, the sintering step improves the electromechanical bonding of the particles [45]. The obtained structures were fixed between two pairs of copper connector wires, which were made in our laboratory to measure sensor response.

#### 2.2.1. Optical Microscopy

**Figure 5:**
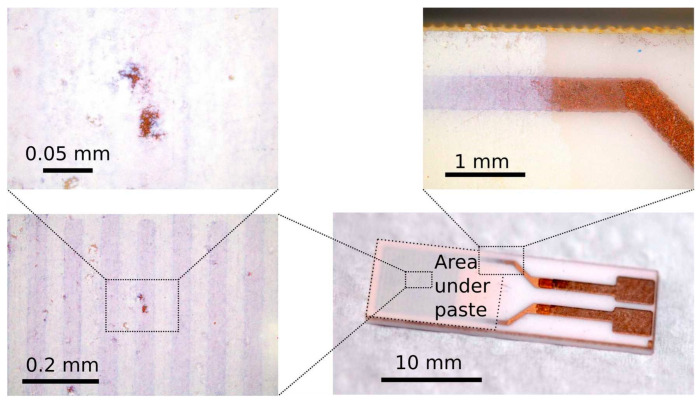
Optical microscopy of the sensor based on the TiCe-0.8:0.2-40 mixture.

Visual inspection of the substrate coverage with the screen-printed material was performed using an optical microscope (BX53MTRF-S, Olympus, Tokyo, Japan). The surface was examined at various points of interest, zooming into the small surface (Figure 5). The optical inspection revealed the uniformity of the screen-print deposition with visible patches of paste of relatively low thickness. This might be the consequence of the manual screen-printing technique used in the process of sensor fabrication.

**Figure 6:**
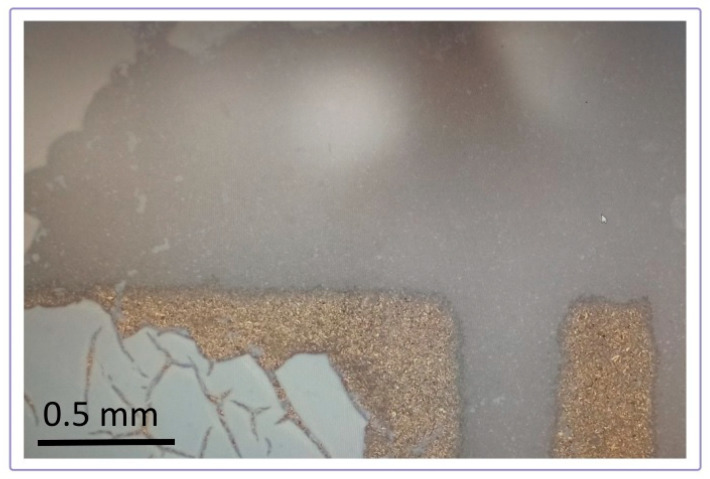
Peeling off of the TiO_2_ layer from the substrate edges.

The thick film of mixtures and CeO_2_ exhibited good attachment to the alumina substrate, without peeling off. On the other hand, it was observed that parts of the TiO_2_ films near the edge of the substrate cracked and fell off after the heat treatment. Figure 6 shows a close examination of the substrate coverage with TiO_2_ films. Parts of the alumina and gold electrodes at the edge of the substrate that are not covered with a film are visible.

#### 2.2.2. Scanning Electron Microscopy of a Film Surface

**Figure 7:**
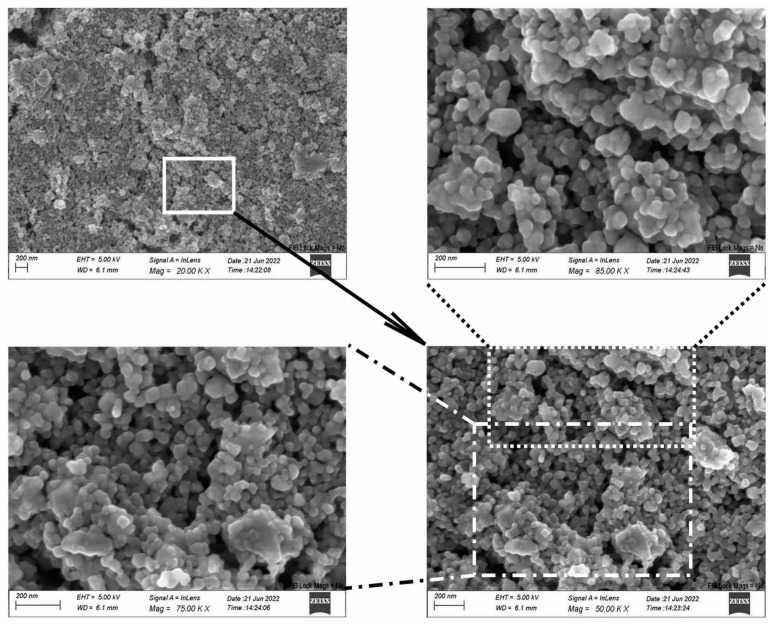
SEM image of the TiCe-0.5:0.5-40 sample at the center of the paste region.

**Figure 8:**
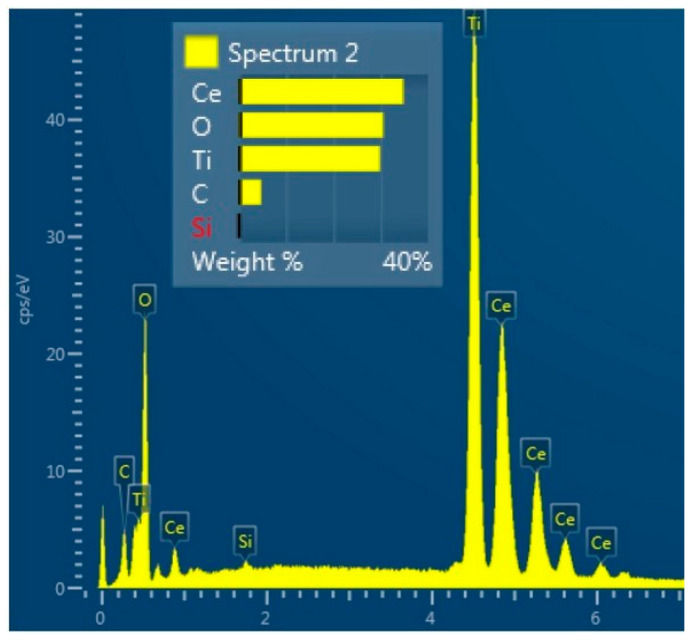
The EDS analysis of the TiCe-0.5:0.5-40 sample.

Scanning Electron Microscopy and Energy Dispersive Spectroscopy (DSM 962, Zeiss, Jena, Germany) were employed to examine the surface of the TiO_2_-CeO_2_ films on the substrate. SEM imaging of the mixtures revealed randomly distributed particles of various size (Figures S1–S5). Small, isolated grains, as well as agglomerates, were observed. Larger particles are irregular in shape, whereas smaller ones appear to be spherical. Such a morphology is a consequence of the wide distribution of particle size of the powder. Identical surface characteristics and a lack of particle ordering were found for all mixtures. The surface structure of the TiCe-0.5:0.5-40 film is given in Figure 7 as an example of a SEM visualization results. EDS analysis showed that the amount of Ti and Ce on the surface of the mentioned sample corresponds to the ratios of the starting oxides (Figure 8). A small amount of carbon is present on the surface of each sample, which is considered an unavoidable contaminant when the material is in contact with air.

#### 2.2.3. Atomic Force Microscopy

**Figure 9:**
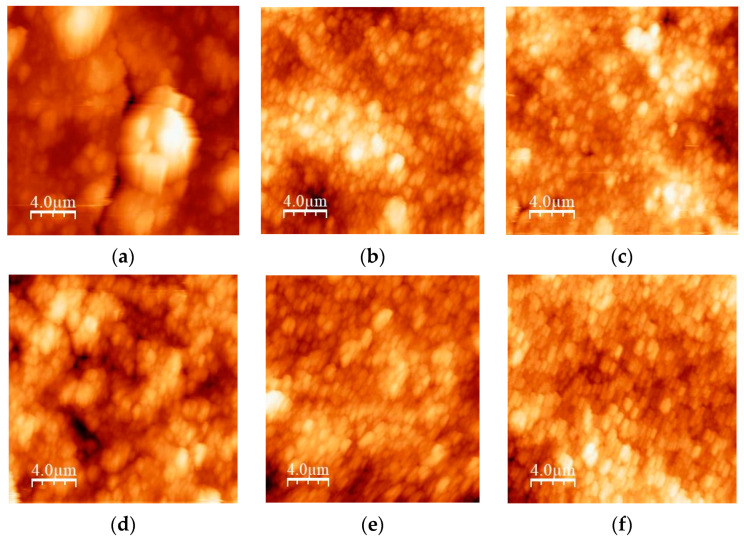
2D AFM images of: (**a**) TiCe-0.8:0.2-40; (**b**) TiCe-0.2:0.8-40; (**c**) TiCe-0.5:0.5-40; (**d**) TiCe-0.8:0.2-100; (**e**) TiCe-0.2:0.8-100; (**f**) TiCe-0.5:0.5-100.

The topography of the thick films was investigated using an atomic force microscope in the intermittent-contact mode (NTEGRA Prima, NT-MDT, Moscow, Russia). The scan area was (20 × 20) µm^2^. The image analysis and roughness parameters calculation were performed using the WSXM 4.0 software beta 9.3 version [46]. In Figure 9a–f, the 2D AFM images for all studied samples are given. The values of the average roughness (R_a_) and the root mean square roughness (R_q_) determined by the mentioned software are given in Table 5. No correlation between roughness and particle size distribution was established. The roughness of the film surface did not change significantly after increasing the milling time, except for the mixture with a high amount of TiO_2_ (TiO_2_:CeO_2_ = 0.8:0.2).

#### 2.2.4. Film Profilometry

**Figure 10:**
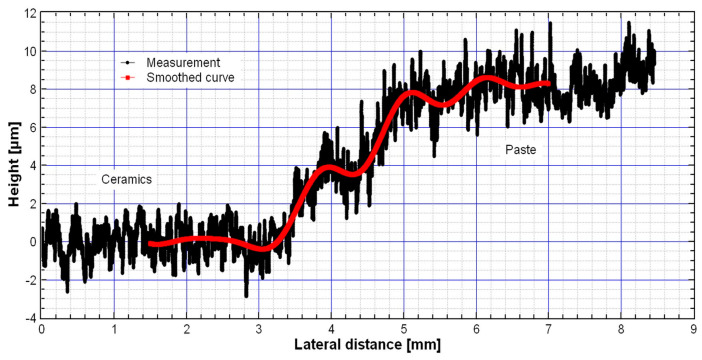
Thickness measurement of the TiCe-0.5:0.5-40 film on the substrate.

The thickness of the thermally treated TiO_2_-CeO_2_ films on the DropSense substrate was measured with a stylus profilometer (DektakXT+N-lite, Bruker, Billerica, MA USA). The force was adjusted to 0.3 mg in order to prevent the film from cutting as the needle of the stylus crosses the surface. Figure 10 shows the thickness measurements result for the sample TiCe-0.5:0.5-40. The layer thickness on the substrate was estimated at 8 µm. A high roughness of the film surface originates by both the non-uniform size of particles and holes in the screen-printing image.

#### 2.2.5. Raman Spectroscopy

The Raman spectra of the films of starting oxides and TiO_2_-CeO_2_ mixtures were recorded with a DXR Raman microscope (Thermo Fisher Scientific, Madison, WI USA), equipped with a research optical microscope and a CCD detector. A frequency-stabilized single mode diode laser with an excitation wavelength of 532 nm was used. The laser power level was adjusted to 1 mW.

**Figure 11:**
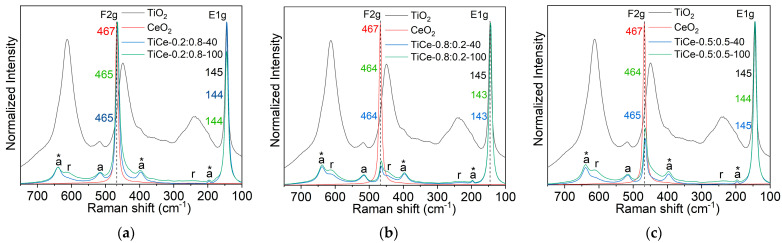
Raman spectrum of: (**a**) TiCe-0.8:0.2-40 and TiCe-0.8:0.2-100; (**b**) TiCe-0.2:0.8-40 and TiCe-0.2:0.8-100; (**c**) TiCe-0.5:0.5-40 TiCe-0.5:0.5-100; the anatase and rutile bands are marked with “a“ and “r“, respectively. The spectra of mixtures of the same composition are arranged in the same graph.

The Raman spectra of the TiO_2_-CeO_2_ films are composed of the characteristic bands of the starting oxides suggesting that a mechanical mixture of oxides was sustainable after thermal treatment (Figure 11a–c). There is no evidence of the formation of detectable compounds between CeO_2_ and TiO_2_.

In the spectra of TiO_2_, two bands belonging to the anatase phase were identified; the most prominent was (E_1g_) [47], and the second one was at 518 cm^−1^ (A_1g_) [47]. The phase transformation of anatase into rutile caused by the thermal treatment of a paste at elevated temperatures [48,49] resulted in the masking of the anatase modes by strong rutile bands. After the mechanochemical activation of mixtures, the appearance and position of the TiO_2_ and CeO_2_ bands changed. The TiO_2_-CeO_2_ spectra revealed three extra bands at around 640, 397, and 195 cm^−1^, assigned to E_3g_, B_1g_, and E_2g_ modes of the anatase phase (marked with an asterisk in Figure 11a–c) [47]. Calculations of Ti-O length relations show that these vibrations correspond to a moderately distorted TiO_6_^−8^ octahedron in anatase [50]. The sharp and narrow features at around 145 cm^−1^ and 464 cm^−1^ indicate high crystallinity of the anatase and CeO_2_ phases in all the synthesized mixtures. Compared to the native TiO_2_, the relative amount of rutile in TiO_2_-CeO_2_ films decreases, as evidenced by the normalized intensity decrease of the Raman bands at 612, 445, and 238 cm^−1^ [50]. In the spectra of the mixtures with the highest amount of CeO_2_ (TiCe-0.2:0.8-40 and TiCe-0.2:0.8-100), anatase vibrations can be clearly seen, whereas the normalized intensity of the rutile band was found to be the lowest among all samples. This leads to the conclusion that the presence of CeO_2_ hinders the anatase-rutile transition, stabilizes the anatase phase, and prevents the growth of the rutile crystals [51]. It can be seen that the characteristic bands of rutile are stronger in the spectra of the mixtures that were subjected to the prolonged milling of 100 min. This coincides with the results of XRD powder analysis, which demonstrated that the rutile weight fraction in mixtures increases with extended milling time (Table 4).

The inhibition of phase transformation most likely occurred due to the formation of the Ce−O−Ti interaction at the interface between the TiO_2_ and CeO_2_ domains [51]. The lattice mismatch between two dissimilar crystals in contact usually causes lattice strain and distortion. The observed shift of the TiO_2_ and CeO_2_ dominant features can be a result of introduced bond distortion [52]. This red shift is more pronounced for the F2g mode, especially in the spectra of samples that do not contain excess CeO_2_, namely: TiCe-0.8:0.2-40, TiCe-0.8:0.2-100, and TiCe-0.5:0.5-100 (up to 3 cm^−1^). Another contribution to the noticeable redshift of the CeO_2_ vibration in the spectra of the mixtures may come from certain concentrations of oxygen vacancies [53] introduced by mechanical forces.

## 3. Results of Electrical Measurements

**Figure 12:**
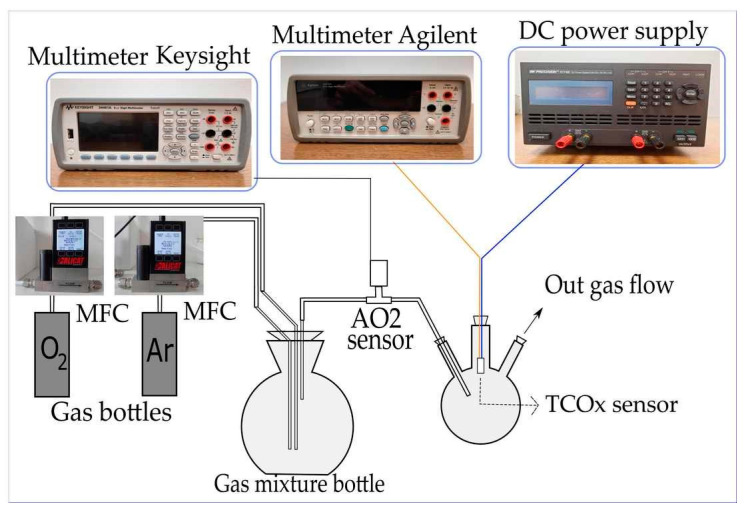
Scheme of the electrical measurements set-up.

Electrical measurements of the obtained structures were performed in order to explore the sensitivity of TiO_2_-CeO_2_ mechanochemically treated mixtures. A system for measurements of the responsiveness of the sensors in gas flow was constructed (Figure 12). The sensors were exposed to different volumetric concentrations of O_2_ in the range from 0 to 100%. The concentration was varied by adjusting the rate of O_2_ flow (5N purity) and Ar (5N purity) using two mass flow controllers (MC-200SCCM-D/5M, Alicat Scientific, Tucson, AZ USA). The total flow of the gas mixture remained constant during the experiment (200 mL/min), but the ratio of O_2_ to Ar flow was altered in order to obtain various O_2_ concentrations. The O_2_ concentration was monitored by measuring the response of the reference sensor (AA428-210 (AO2) Citicel, Honeywell City Technology, India) using a multimeter (34461A, Keysight, Santa rosa, CA USA). The TiO_2_-CeO_2_ (TCOx) sensor was placed in a closed glass flask with inlets for the gas mixture and electrical contacts for the sensor. The sensor was heated by connecting the built-in platinum heater to a direct current (DC) power supply (9174B, B&K Precision, Yorba Linda, CA USA).

The supply voltage level was adjusted to 7 V, 8 V, and 9 V to obtain different working temperatures of the TCOx sensor (270 °C, 300 °C, and 320 °C, respectively). After setting the voltage, the value of the DC supply current was monitored. A stable current was taken as an indicator of a steady temperature. The temperature of the TCOx sensor was previously calibrated using an IR thermal camera (E5, FLIR Systems, Wilsonville, OR USA). Direct current resistance (DCR) measurements of the sensors were performed using a multimeter (34410A, Agilent, Santa Clara, CA USA), as it has the capability of measuring the resistance up to 1 GΩ.

**Figure 13:**
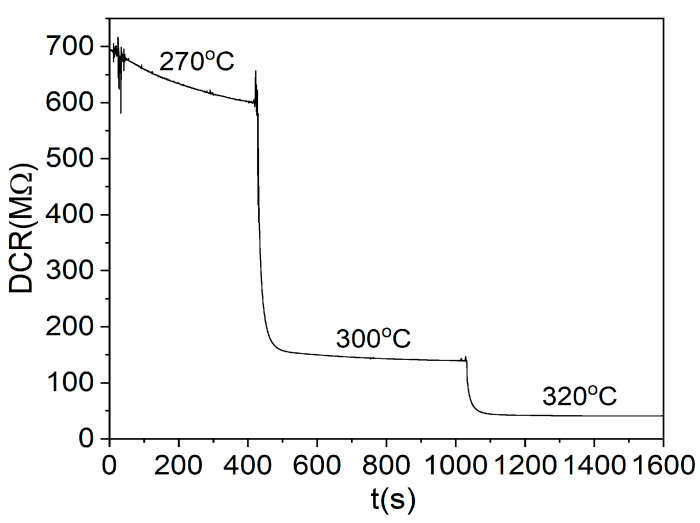
Dependence of the DC resistance of the TCOx sensor on temperature at 30% O_2_ volumetric concentration for the TiCe-0.5:0.5-40 sample.

The TCOx sensors were tested at different temperatures for a fixed O_2_ concentration. The same temperature behavior was observed for all mixtures. The minimum temperature at which the resistance falls below 1 GΩ and becomes measurable was around 270 °C. As the temperature rises, the resistance drops sharply to lower levels. As an example of the measurements at different temperatures, the response of the sensor based on the TiCe-0.5:0.5-40 mixture is shown in the Figure 13.

**Figure 14:**
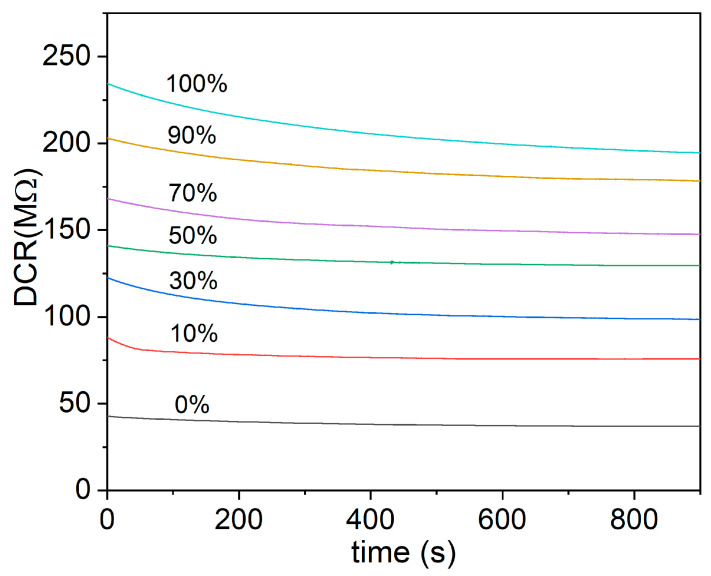
An example of the time monitoring of the resistance at different volumetric concentrations of O_2_ for the TiCe-0.8:0.2-100 mixture (this sample is not shown in the Figure 15).

The TCOx sensor response measurements presented in this study were carried out at 320 °C. This operating temperature was chosen as the minimum temperature at which resistance of the sensor is low enough to be measurable by most available instruments for all samples. The resistance values were monitored for 15 min to test the stability of the sensor response at 320 °C. Figure 14 shows the results of time-dependent electrical measurement for the TiCe-0.8:0.2-100 mixture at a given operating temperature.

**Figure 15:**
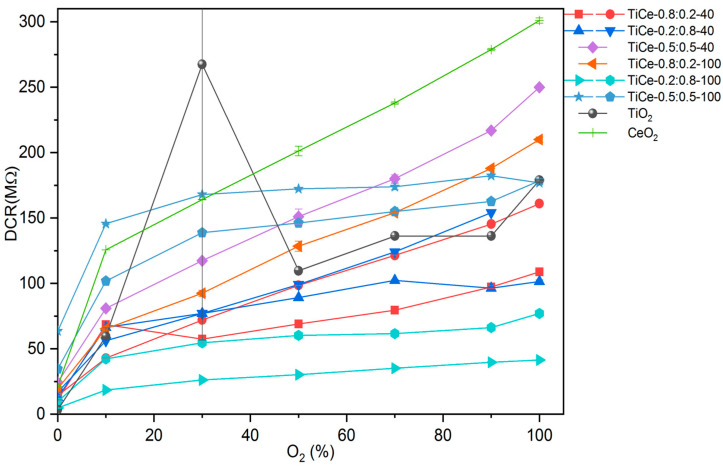
Measurements of the resistance of the mixed and pure oxides at different volumetric concentrations of O_2_ at a temperature of 320 °C.

The mean value along with the standard deviation was derived from the last 200 s of electrical measurement for all TCOx sensors and presented in Figure 15 as the dependence of DC resistance on O_2_ volumetric concentration. Some of the oxide mixtures were screen printed on two substrates in order to test the repeatability of the sensing layer fabrication process. The same color on the graph indicates TCOx sensors made out of the same mixture.

It can be seen that the pure TiO_2_ film shows a very erratic response to the O_2_ concentrations, with a large standard deviation at 30% O_2_. We assume that the main cause of the unreliable sensor response is the peeling of the TiO_2_ film from the edge of the substrate, which is observed after heat treatment. For samples containing some amount of CeO_2_, such an irregular response of the sensor was not found, which can be seen in the behavior of other sensors, except the one based on pure TiO_2_. Most of the samples show monotonic behavior, with resistance increasing from low values to significantly higher values as the O_2_ concentration was raised. This feature makes the particular sample useful in real-world applications, since the correspondence between measured resistance and oxygen concentration is unambiguous. The resistance of the screen-printed CeO_2_ film is relatively high, typically on the order of hundreds of MΩ. This makes the practical applicability of the CeO_2_ sensor challenging, since it is hard to measure the resistance of such a range. Electrical measurements have shown that TiO_2_-CeO_2_ films have significantly lower resistance than pure CeO_2_. From the data in Figure 15, we found that the mixture TiCe-0.2:0.8-100 has the lowest absolute value of resistance.

Since the used screen printing equipment is manual, parameters such as the pressure on the screen and the amount of paste applied can vary with each deposition of a layer. The insufficient reproducibility of the screen printing technique obviously affected the reproducibility of sensor performance (Figure 15). The oxygen responses of sensors based on the identical TiO_2_-CeO_2_ mixtures were slightly different. This discrepancy can also originate from random errors introduced during the paste preparation. Moreover, the wide particle size distribution of the mechanochemically synthesized powders can contribute to the non-uniformity of the screen-printed layers. However, it was observed that the resistance values of the same TCOx sensors are grouped next to each other. In order to achieve the best possible reliable oxygen response, optimization of the screen printing process is required.

**Figure 16:**
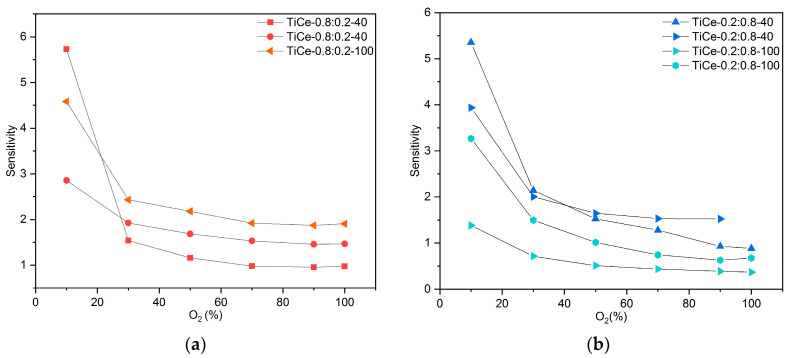

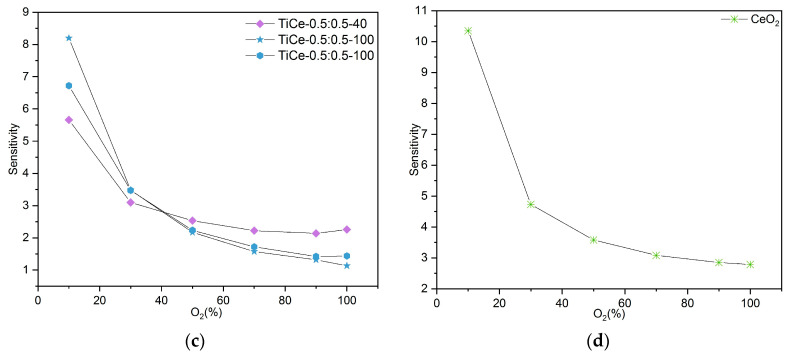
Obtained values for sensitivity of: (**a**) mixtures with ratio TiO_2_:CeO_2_ =0.8:0.2; (**b**) mixtures with ratio TiO_2_:CeO_2_ = 0.2:0.8; (**c**) mixtures with ratio TiO_2_:CeO_2_ = 0.5:0.5; (**d**) CeO_2_.

The sensitivity of the TCOx sensor was calculated as the ratio between the change in resistance and the change in oxygen concentration:

S = ΔR/ΔO_2_ (3)

where S is the sensitivity, ΔR is the resistance change, and ΔO_2_ is the change in oxygen concentration. The obtained values for the sensitivity of CeO_2_ and TCOx sensors are presented in Figure 16a–d. Results for mixtures of the same composition are shown in the same graph.

According to the graph data, the sensitivity of all mixtures strongly depends on oxygen level; it is the highest in the region of low concentrations (around 10%) and then drops sharply. The mixtures of composition TiO_2_:CeO_2_ = 0.5:0.5 that were milled for 100 min proved to be the most sensitive to the presence of oxygen (Figure 16c). The highest sensitivity was obtained in the range from 10% to 30%, reaching values between 3.5 and 8.2. In the case of mixed oxides at the mass ratio TiO_2_:CeO_2_ = 0.2:0.8, a trend of decreasing sensitivity with prolonged milling time was observed. Although the sensitivity of the sensor based on CeO_2_ is higher than that of the examined mixtures, its resistance exceeds 100 MOhm, even for very low percentages of O_2_.

## 4. Discussion

**Figure 17:**
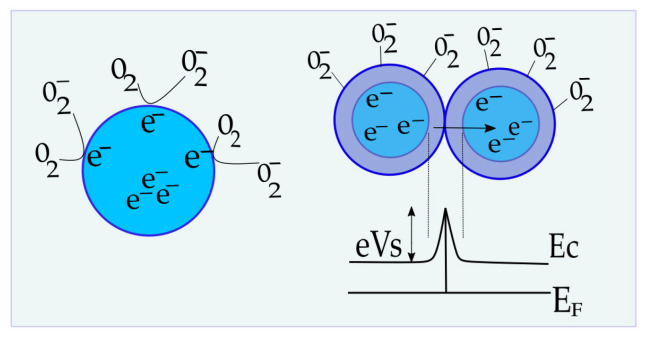
The illustration on the left is the receptor function of the semiconductor; right is the transducer function, band bending, and Schottky barrier between the same adjacent grains.

The sensing mechanism of semiconductor gas sensors is based on a shift of equilibrium of the surface oxygen chemisorption as a consequence of the change in environmental oxygen concentration [6,54]. The sensing properties are assumed to be influenced by two functions: receptor function and transducer function (Figure 17) [6]. According to Korotcenkov and Cho [55], receptor function represents the ability of the metal oxide surface to interact with the target gas. Therefore, it is determined by the chemical properties of surface oxygen. When the surface of the sensing layer is changed by adding a noble metal or another oxide, the modification of receptor function occurs [6]. The sensitivity can be significantly affected in this way. The second function determines the efficiency of oxygen-oxide interactions’ detection and conversion into an electrical signal [56]. It is based on the free carrier transport mechanism through the barrier between adjacent grains, which takes place at high temperatures. Contact between identical neighboring grains is called homo-contact [57]. A double Schottky barrier model is most often used to describe the free carrier transport process [6,56].

When O_2_ molecules are adsorbed on an n-type oxide surface, they extract electrons from the conduction band (Ec) and trap them at the surface. Double charged oxygen vacancies (V^••^) are the dominant active sites that O_2_ occupies, and they behave as electron donors [6]:

V^••^ + 2e^−^ + 1/2O_2_→O_ionic_ (4)

As a result of surface occupation by O_2_, the metal oxide layer loses electrons and ionic species are formed (O_ionic_ = O_2_^−^, O^−^, O^2−^) [18]. O^−^ is believed to be the dominant form of adsorbed oxygen at the operating temperature of 300–450 °C [56]. In addition, the layer of charged oxygen species on the surface repels other electrons from the bulk of films, causing the formation of an electron-depleted region, and hence increasing the height of the Schottky barrier (eV_s_ on Figure 16) [6]. The thickness of the electron-depleted region, the so-called space-charge layer, is the length of the band bending region. The creation of a potential barrier at the grain boundary restricts the flow of electrons and increases the resistance of the sensing layer [6,58].

As a result of the chemisorption of oxygen molecules, electron mobility and active sites for further adsorption are reduced. The electrical conductivity of the n-type semiconductor decreases with increasing oxygen partial pressure (p_O_), following the equation [6]:

σ = Aexp(−E_A_/kT)p_O_^1/N^ (5)

where σ is the conductivity, A is the constant, E_A_ is the activation energy that determines adsorption reaction rate, k is the equilibrium constant, T is the working temperature, and N is the value that depends on the type of the defect. For the n-type semiconductor, this value is negative. Observing the calibration curve in Figure 14, it becomes clear that the TiO_2_-CeO_2_ mixtures behave as individual n-type oxides over the whole range of partial pressure, as well as pure CeO_2_.

Raman spectroscopy of the screen-printed TiO_2_-CeO_2_ films revealed that CeO_2_ crystals surround TiO_2_ crystals, inhibiting the anatase-rutile phase transformation. This implies that the TiO_2_ and CeO_2_ domains are in direct contact, forming a defined hetero-interface. Although the understanding of the working mechanism of sensors based on mixed oxides is still far from satisfactory, it is well known that the hetero-interface largely affects the sensing properties. The nature of the oxide-oxide interface can be briefly described with the help of characterization methods. XRD spectra of TiO_2_-CeO_2_ powders indicate that no solid solution between TiO_2_ and CeO_2_ was formed, at least not in a detectable amount. The Raman spectroscopy of the thick films did not show any features attributable to the new compound (Figure 11a–c), further indicating that the heat treatment of the paste did not lead to chemical bonding between TiO_2_ and CeO_2_. Thus, it is most likely that the interaction at the TiO_2_-CeO_2_ interface is physical. The red shift of the CeO_2_ band in the Raman spectra further suggests that the presence of the interface facilitates the reduction of Ce^4+^ ions and hence the formation of oxygen vacancies in mixtures. Studies that confirm this through experimental or theoretical analysis can be found [59,60].

When assembling two types of semiconductors with significantly different physical properties and work functions, in addition to homo-contacts, hetero-contacts are formed [57]. A hetero-contact is an interface between two types of grains, characterized by the generation of a potential that represents a thin double layer of physically separated positive and negative charges. The contact potential attenuates drift mobility for the electrons running in the direction against it, although it does not affect the electrons running in the opposite direction, hence increasing the resistivity of the interface. As a result, electrons with insufficient energy to overcome the contact potential are scattered, and the resistance of the material increases. Hetero-contacts can have a drastic impact on the sensor transducer’s function if the structure of the sensing layer is properly designed [61]. In that case, the response of the sensor is affected by the presence of the target gas in two ways: by changing the surface density of grain electrons and by changing the drift mobility of electrons passing through the contact. High sensing performances can be achieved by selecting the appropriate combination of grains and the structure in which the grains are packaged [57,61].

SEM imaging of the surface of TiO_2_-CeO_2_ films revealed irregularly distributed grains (particles) of different sizes with a tendency to form agglomerates (Figure 7). Grains that are randomly packed into a three-dimensional structure are considered a random network of homo- and hetero-contact potential [57]. N. Yamazoea and K. Shimanoea [61] showed that the resistance of the semiconductor structure is almost cut in half when grains of two different semiconductors are randomly mixed together, independent of the presence of the target gas. All particles are assumed to be the same size. Such a chaotically arranged structure will allow current to flow through a path of lower resistance contact, such as homo-contacts. Hetero-contacts will not act as the main electron transport pathway [57]. Therefore, simply mixing two groups of semiconductors is not an appropriate structure design, as it contributes to its conductivity. Electrical measurements performed in this study revealed that the most noticeable change in the response of TCOx sensors compared to pure CeO_2_ is precisely the lower value of electrical resistance for each oxygen concentration. TiO_2_ is excluded from the discussion due to its unreliable response to oxygen.

Since the particle size distribution obtained by high-energy ball milling is wide (Figure 2a–e), the approximation of grains of the same size is not well-founded for this study. The contribution of hetero-contact between grains of different sizes cannot be neglected. XRD analysis of the powders showed a significant amount of the rutile phase induced by mechanochemical forces (Table 4). Hence, better O_2_ adsorption and its more efficient reduction to the rutile phase then to the anatase should also be taken into account [62]. Increasing the amount of rutile in the mixture can improve the sensitivity of the sensor. The coexistence of the three phases, anatase, rutile, and CeO_2_, and their interactions, determine the structure of TiO_2_-CeO_2_ in a complex and uncertain way, which makes it demanding to establish a specific relation that would describe the influence of milling parameters on sensing properties of the TCOx sensor.

Like it was already mentioned, receptor function is concerned with the surface structure of the sensing material. Based on the AFM imaging results of the film surface, the receptor function of TCOx sensors does not appear to alter much (Table 5). A significantly higher parameter of roughness for the mixture TiCe-0.8:0.2-40 deviates from the others, which are more or less similar. With an increase in the milling time during the synthesis of TiCe-0.8:0.2-40 powder, the surface area of the film became significantly smoother. Most interestingly, the film with the smallest surface roughness was the one made from mixtures with a ratio of TiO_2_:CeO_2_ = 0.5:0.5. Just such a mixture milled for 100 min showed the highest sensitivity to oxygen among the tested mixtures.

## 5. Conclusions

The purpose of this study was to show that the mechanochemical process can be used to create a TiO_2_-CeO_2_ powder mixture suitable for oxygen sensors. The mechanochemical synthesis in conjunction with screen printing was successful, generally indicating the potential application of this route for the fabrication of sensors for a variety of purposes. The procedure in a high-energy planetary ball mill corresponds to the green chemistry trend of avoiding or minimizing the use of solvents, increasing selectivity toward the synthesis of a specific product, and reducing the number of synthesis steps.

The research presented in this paper also shows that there is plenty of room for improvement in both the powder synthesis procedure and the procedure for producing screen printing paste with improved adhesion properties. The synthesis conditions did not provide the ideal structure of the sensing material, as demonstrated in the paper. Thin films of TiO_2_-CeO_2_ are mechanical mixtures with randomly distributed homo- and hetero-contacts. Nonetheless, electrical measurements show that the mechanochemically treated TiO_2_-CeO_2_ mixed oxides have promising properties for oxygen sensing at lower working temperatures than the metal oxide-based sensor. The mixture of TiO_2_-CeO_2_ in the ratio 0.2:0.8, ground for 100 min, has the lowest absolute resistance, which is advantageous in terms of electrical measurement simplicity. This study also reveals that TiO_2_ films have poor adhesion to an alumina substrate and an erratic response to oxygen levels. With the addition of CeO_2_, adhesion and oxygen responsiveness improve significantly. The study of the electronic structure of the mixtures will be one of the primary focal points of future research. We anticipate that this will help to provide a more accurate description of the sensing mechanism of TCOx sensors. Work will also be carried out on the improvement of the paste screen printing process and the optimization of mechanochemical synthesis parameters.

## Figures and Tables

**Table 1:** Labelling of the samples after mechanochemical treatment.

Sample	Mass Ratio (TiO_2_:CeO_2_)	Milling Time
TiCe-0.8:0.2-40	0.8:0.2	40 min
TiCe-0.2:0.8-40	0.2:0.8	40 min
TiCe-0.5:0.5-40	0.5:0.5	40 min
TiCe-0.8:0.2-100	0.8:0.2	100 min
TiCe-0.2:0.8-100	0.2:0.8	100 min
TiCe-0.5:0.5-100	0.5:0.5	100 min

**Table 2:** The particle size values at 10%, 50%, and 90% of the cumulative frequency and span values.

Sample	d_10_ [µm]	d_50_ [µm]	d_90_ [µm]	Span
TiO_2_	1.25	3.70	10.61	2.532
CeO_2_	3.80	22.58	55.81	2.303
TiCe-0.8:0.2-40	0.96	7.88	33.79	4.166
TiCe-0.2:0.8-40	0.52	6.72	29.27	4.282
TiCe-0.5:0.5-40	0.74	8.08	39.9	4.845
TiCe-0.8:0.2-100	0.80	7.97	29.56	3.609
TiCe-0.2:0.8-100	0.52	5.01	18.66	3.618
TiCe-0.5:0.5-100	0.55	5.50	20.54	3.635

**Table 3:** The crystallite size of anatase, rutile, and ceria in mixtures and pure oxides.

Sample	Anatase (nm)	Rutile (nm)	Ceria (nm)
TiCe-0.8:0.2-40	20.65	27.38	46.04
TiCe-0.2:0.8-40	12.97	-	33.32
TiCe-0.5:0.5-40	17.87	33.19	41.98
TiCe-0.8:0.2-100	18.98	30.40	39.28
TiCe-0.2:0.8-100	-	-	24.97
TiCe-0.5:0.5-100	15.93	26.86	31.25
TiO_2_	21.48	31.52	-
CeO_2_	-	-	49.50

**Table 4:** The weight fraction of rutile in the mixtures and starting TiO_2_.

Sample	Rutile Weight Fraction
TiCe-0.8:0.2-40	0.27
TiCe-0.2:0.8-40	-
TiCe-0.5:0.5-40	0.36
TiCe-0.8-0.2-100	0.35
TiCe-0.2:0.8-100	-
TiCe-0.5:0.5-100	0.40
TiO_2_	0.19

**Table 5:** Roughness parameters estimated from AFM measurements.

Sample	R_a_ (nm)	R_q_ (nm)
TiCe-0.8:0.2-40	420	560
TiCe-0.2:0.8-40	180	220
TiCe-0.5:0.5-40	130	170
TiCe-0.8:0.2-100	190	230
TiCe-0.2:0.8-100	170	210
TiCe-0.5:0.5-100	120	140

## Data Availability

Data available upon request.

## Supplementary Materials

The following supporting information can be downloaded at: https://www.mdpi.com/article/10.3390/s23031313/s1, Figure S1: SEM image of the TiCe-0.8:0.2-40 sample at the center of the paste region; Figure S2: SEM image of the TiCe-0.2:0.8-40 sample at the center of the paste region; Figure S3: SEM image of the TiCe-0.8:0.2-100 sample at the center of the paste region; Figure S4: SEM image of the TiCe-0.2:0.8-100 sample at the center of the paste region; Figure S5: SEM image of the TiCe-0.5:0.5-100 sample at the center of the paste region.
