# Supplementary material for: Mechanochemical Synthesis of TiO2-CeO2 Mixed Oxides Utilized as a Screen-Printed Sensing Material for Oxygen Sensor
Source: Sensors (Basel). 2023 Jan 24;23(3):1313. doi: 10.3390/s23031313 (PMC9919251; doi:10.3390/s23031313)
Supplement: Supplementary file 1 [file sensors-23-01313-s001.zip › sensors-2141711-supplementary.pdf]

Supplementary Materials

2.2.2. Scanning Electron Microscopy of a film surface

The following SEM images were acquired using a Hitachi Regulus 8220 Scanning Electron Microscope (Mito, Japan).

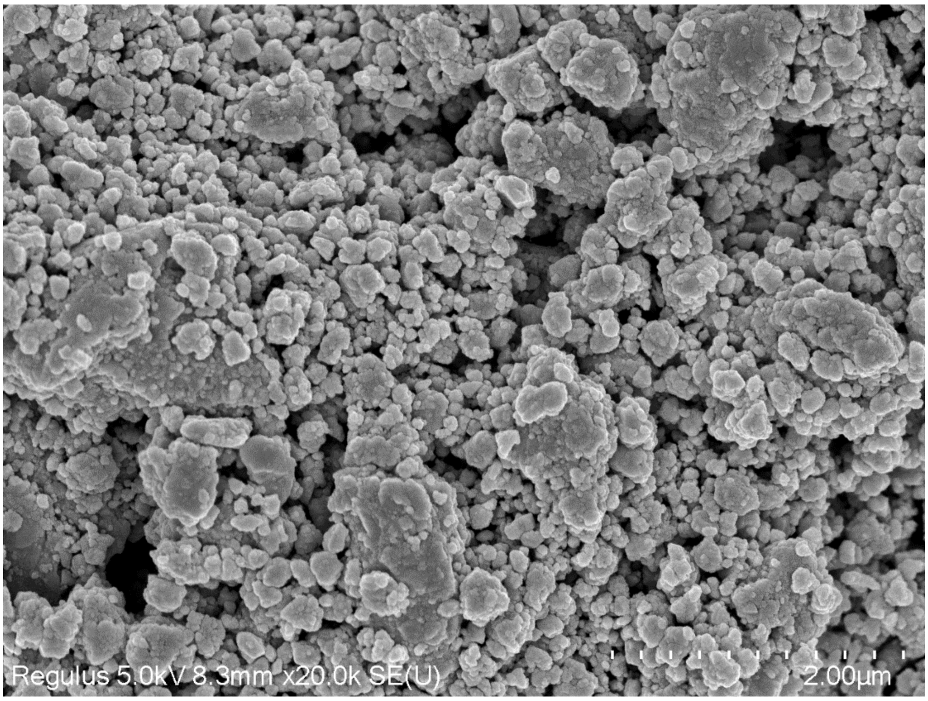

Figure S1. SEM image of the TiCe-0.8:0.2-40 sample at the center of the paste region.

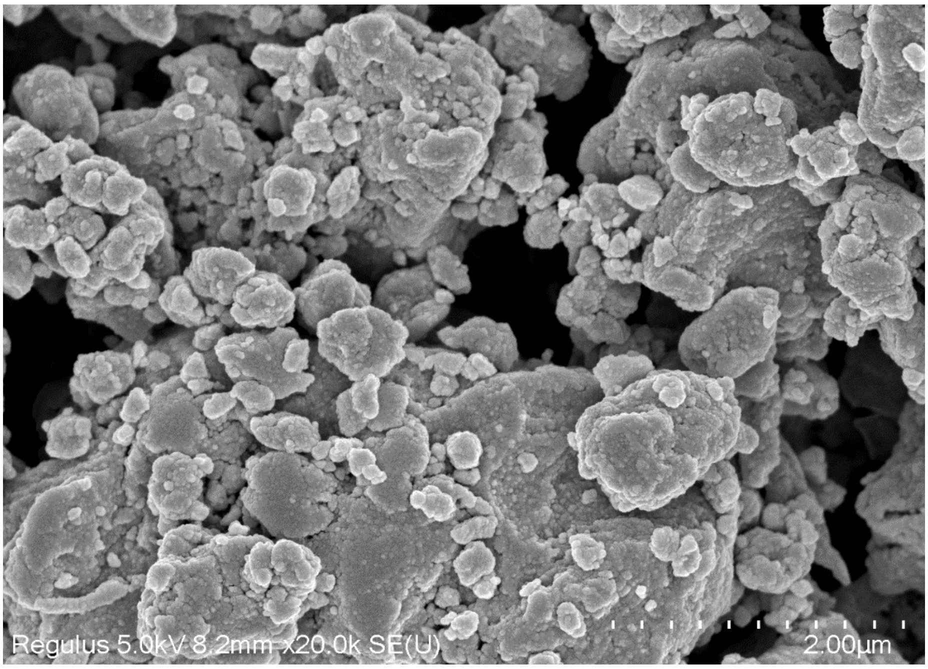

Figure S2. SEM image of the TiCe-0.2:0.8-40 sample at the center of the paste region.

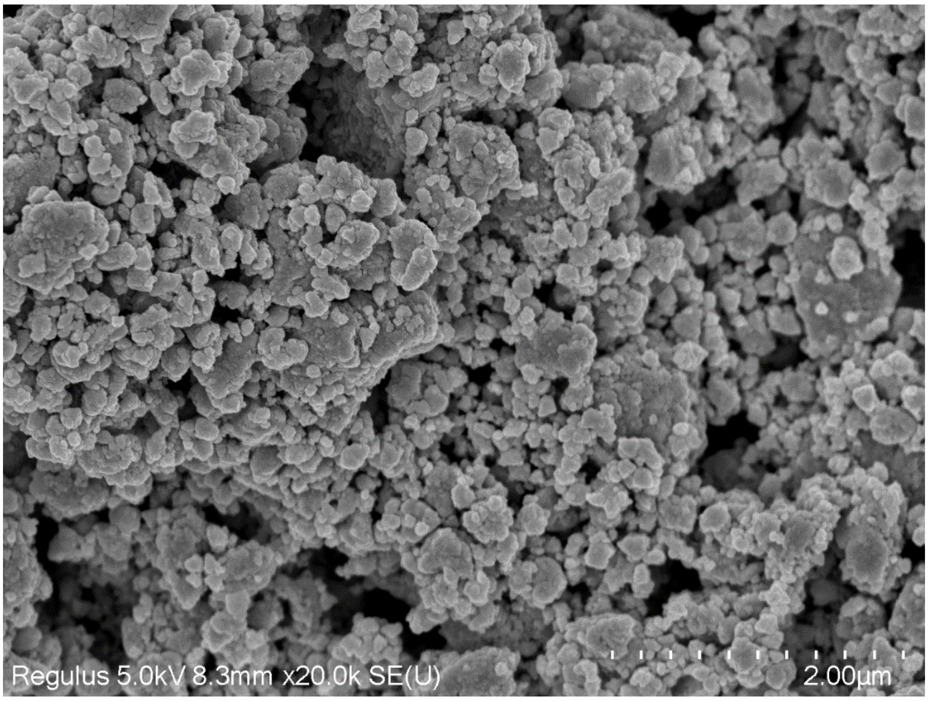

Figure S3. SEM image of the TiCe-0.8:0.2-100 sample at the center of the paste region.

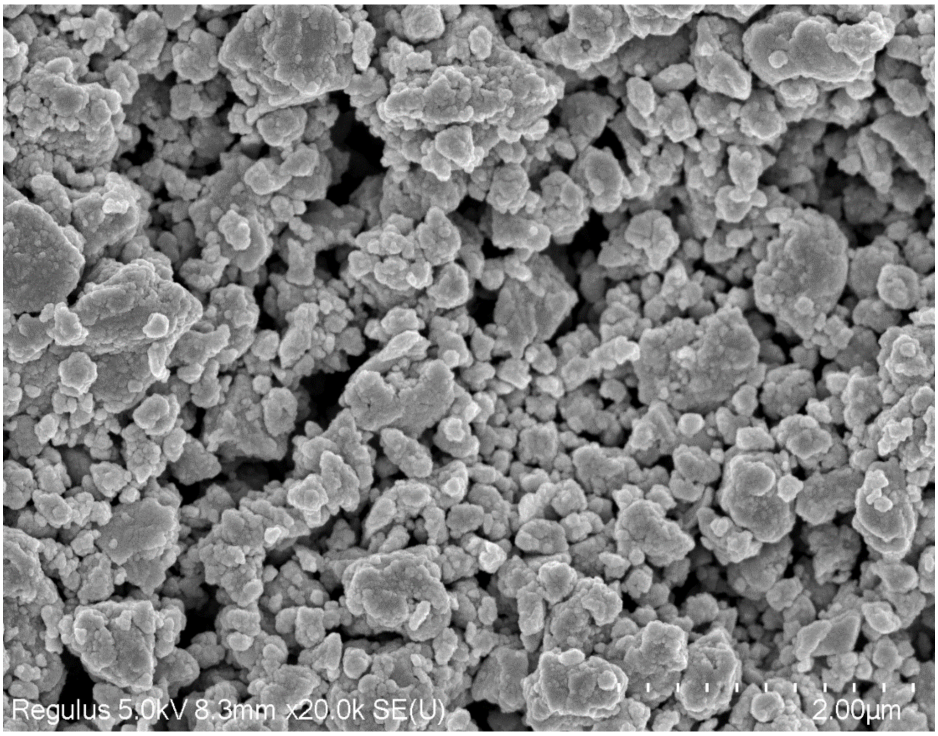

Figure S4. SEM image of the TiCe-0.2:0.8-100 sample at the center of the paste region.

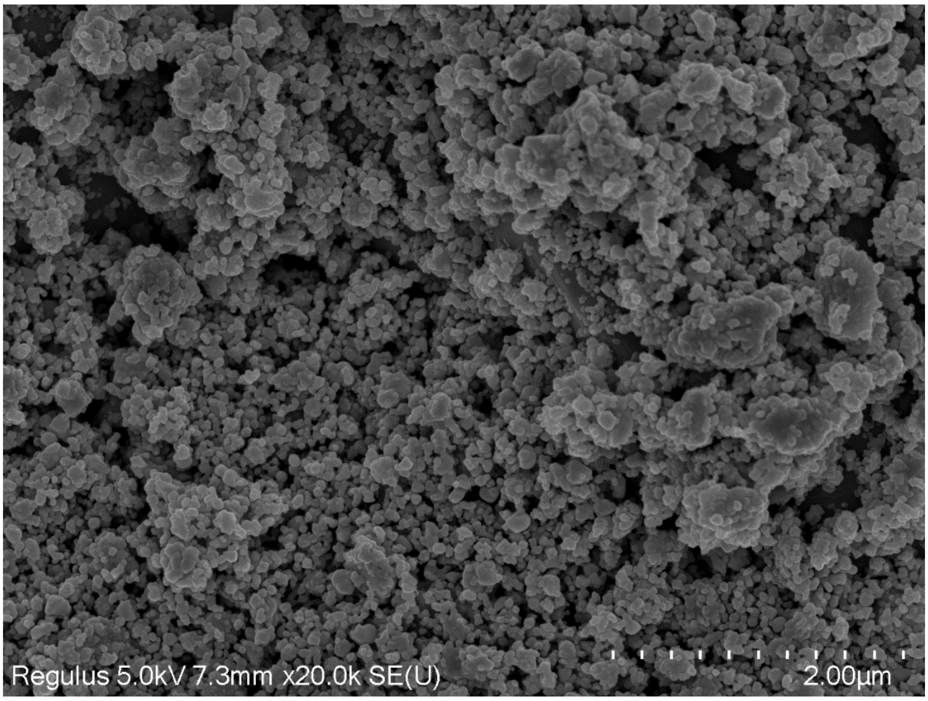

Figure S5. SEM image of the TiCe-0.5:0.5-100 sample at the center of the paste region.
